# Effect of Carotenoids, Oligosaccharides and Anthocyanins on Growth Performance, Immunological Parameters and Intestinal Morphology in Broiler Chickens Challenged with *Escherichia coli* Lipopolysaccharide

**DOI:** 10.3390/ani10020347

**Published:** 2020-02-21

**Authors:** Brigitta Csernus, Sándor Biró, László Babinszky, István Komlósi, András Jávor, László Stündl, Judit Remenyik, Péter Bai, János Oláh, Georgina Pesti-Asbóth, Levente Czeglédi

**Affiliations:** 1 Department of Animal Science, Institute of Animal Science, Biotechnology and Nature Conservation, Faculty of Agricultural and Food Sciences and Environmental Management, University of Debrecen, 4032 Debrecen, Hungary; komlosi@agr.unideb.hu; 2 Doctoral School of Animal Science, University of Debrecen, 4032 Debrecen, Hungary; 3 Department of Human Genetics, Institute of Microbiomics, Faculty of Medicine, University of Debrecen, 4032 Debrecen, Hungary; sbiro@med.unideb.hu; 4 Department of Feed and Food Biotechnology, Institute of Animal Science, Biotechnology and Nature Conservation, Faculty of Agricultural and Food Sciences and Environmental Management, University of Debrecen, 4032 Debrecen, Hungary; babinszky@agr.unideb.hu; 5 Department of Laboratory of Animal Genetics, Institute of Animal Science, Biotechnology and Nature Conservation, Faculty of Agricultural and Food Sciences and Environmental Management, University of Debrecen, 4032 Debrecen, Hungary; javor@agr.unideb.hu; 6 Institute of Food Technology, Faculty of Agricultural and Food Sciences and Environmental Management, University of Debrecen, 4032 Debrecen, Hungary; stundl@agr.unideb.hu (L.S.); remenyik@agr.unideb.hu (J.R.); georgina.asboth@agr.unideb.hu (G.P.-A.); 7 Department of Medical Chemistry, Faculty of Medicine, University of Debrecen, 4032 Debrecen, Hungary; baip@med.unideb.hu; 8 Farm and Regional Research Institute of Debrecen, University of Debrecen, 4032 Debrecen, Hungary; olahja@agr.unideb.hu

**Keywords:** natural compounds, β-glucan, carotenoids, oligosaccharides, anthocyanins, broiler chicken, gene expression, cytokines, receptors, intestinal morphology

## Abstract

**Simple Summary:**

In recent years, substitution or reduction of antibiotic application has become a general aim in poultry industry, after concerns about multiresistant bacteria appeared. Accordingly, many natural compounds are used as potential immunostimulants to enhance immune responses. In this research, the effects of carotenoids, oligosaccharides and anthocyanins were investigated on chicken inflammatory cytokines and receptors. Being part of the innate immune system, cytokines are peptides which take part in signaling processes between cells and regulate inflammatory responses. Toll-like receptors are cell surface receptors which bind to antigens specifically. Gene expression levels of some cytokines such as interleukin-1β, interleukin-6, interferon-α, interferon-γ and toll-like receptors such as toll-like receptor 4, toll-like receptor 5 were evaluated in chicken spleen and ileum by Real Time Polymerase Chain Reaction (Real Time PCR) analyses. Relative gene expression level of splenic interleukin-1β decreased in carotenoid-, oligosaccharide- and anthocyanin treated chickens, and relative mRNA level of splenic interleukin-6 was lower in birds fed carotenoid supplement, which could represent a beneficial effect of mentioned natural compounds. Effects of compounds were also examined on gut morphology, where natural agents may result in better absorptive functions.

**Abstract:**

This study was conducted to investigate the effect of carotenoid, oligosaccharide and anthocyanin supplementation in broiler diets under *Escherichia* *coli* lipopolysaccharide (LPS) challenge. Ross 308 chickens were fed 5 diets: basal diet (control diet), diet supplemented with β-glucan in 0.05% (positive control) and diets with 0.5% carotenoid-, oligosaccharide- or anthocyanin contents. On the 26th days of age, chickens were challenged intraperitoneally 2 mg LPS per kg of body weight. 12 h after injection, birds were euthanized, then spleen and ileum samples were collected. LPS induced increased relative mRNA expression of splenic (*p* = 0.0445) and ileal (*p* = 0.0435) interleukin-1β (*IL-1β*), which was lower in the spleen in carotenoid (*p* = 0.0114), oligosaccharide (*p* = 0.0497) and anthocyanin (*p* = 0.0303)-treated chickens compared to LPS-injected control birds. Dietary supplementation of carotenoids also decreased relative gene expression of splenic interleukin-6 (*IL-6*) (*p* = 0.0325). In the ileum, β-glucan supplementation showed lower relative mRNA expression of toll-like receptor 5 (*TLR-5*) (*p* = 0.0387) compared to anthocyanin treatment. Gene expression of both splenic and ileal interferon-α (*IFN-α*), interferon-γ (*IFN-γ*), toll-like receptor 4 (*TLR-4*) and toll-like receptor 5 (*TLR-5*) were not influenced by dietary supplements. In conclusion, carotenoids, oligosaccharides and anthocyanins could partially mitigate the immune stress caused by LPS challenge. All of the compounds impacted longer villus height (*p* < 0.0001), villus height:crypt depth ratios were higher after β-glucan (*p* < 0.0001) and anthocyanin (*p* = 0.0063) supplementations and thickened mucosa was observed in β-glucan (*p* < 0.0001), oligosaccharide (*p* < 0.0001) and anthocyanin (*p* = 0.048) treatments. All of these findings could represent a more effective absorption of nutrients.

## 1. Introduction

Health status and performance of farm animals can be affected by numerous diseases. Among illnesses, bacterial infections of poultry such as salmonellosis and coccidiosis, are prevented by antibiotics [1,2]. Application of antibiotics in agriculture has led to increasing concerns due to resistance in pathogens [3]. Resistant bacteria can be transferred to human population from livestock directly and human health can be also influenced by contaminated foodstuffs. Horizontal gene transfer is another route to receive resistance genes from agriculture into human pathogenic bacteria [4]. Previous studies showed that fluoroquinolone-resistant *Campylobacter* spread by chicken contamination [5], clinical infections caused by resistant zoonotic pathogens, such as *Salmonella* and *Campylobacter*, methicillin-resistant *Staphylococcus aureus* transmission among human population [4] and carbapenem-resistant *Enterobacteriace* with easily transferable resistance gene (NDM) originated from chicken [6] exemplify the prevalence of antibiotic resistance in agriculture, which led to antibiotic regulations worldwide [4].

Therefore, many natural agents have been investigated as potential immunomodulators in recent years to substitute or reduce the amount of antimicrobial drugs [7,8,9]. The immunostimulant impact of β-glucan (yeast cell wall extract) has been proved [10]. Live yeast could reduce levels of pro-inflammatory cytokines as interferon-γ (IFN-γ) and interleukin-1β (IL-1β) levels in pigs [11,12] and increase anti-inflammatory interleukin-10 (*IL-10*) mRNA expression in chicken [13]. Therefore, it is widely used in the poultry industry to improve both the humoral and cellular immune responses and to strengthen the defense system [14]. 

Other possible compounds can be applied in the diet to modulate the immune system by boosting immune responses [15]. Carotenoids are pigments which produce the bright yellow, red and orange colors in plants [16]. These pigments can function as antioxidants and immunomodulators, as well [17]. Some of their compounds, e.g., astaxanthin showed an anti-inflammatory effect in LPS-stimulated mice when expression of pro-inflammatory cytokines, as tumor necrosis factor-α (*TNF-α)* and interleukin-1β (*IL-1β)* were inhibited [18]. Other xanthophylls could also decrease gene expression levels of pro-inflammatory interleukin-1β (*IL-1β*), interleukin-6 (*IL-6*) and interferon-γ (*IFN-γ*) in hens [19]. Oligosaccharides are natural components of vegetable or fruit extracts. Several oligosaccharides, such as fructooligosaccharides, mannan-oligosaccharides or galactooligosaccharides cannot be digested in the intestine of monogastrics. Rather, they reach the colon and are fermented by useful microorganisms [20]. Therefore, these oligosaccharides are often used as prebiotics as they represent natural alternatives to antibiotic growth promoters (AGPs), can enhance production performance and stimulate the immune system [21,22,23,24]. Some of these oligosaccharides, such as fructooligosaccharides and mannan-oligosaccharides are reported to improve intestinal structures as well [25,26,27]. Anthocyanins are a group of flavonoids found in berries to produce the colors red, purple or blue [28]. Today, anthocyanins are being applied as feed components because of their possible antioxidant, anti-inflammatory and immunostimulant properties. Yet, their effects on avians are less known [29]. A few studies have shown that anthocyanin-rich-fragments could inhibit the expressions of pro-inflammatory cytokines in mice [28].

As part of the innate and acquired immunity, cytokines act as extracellular signals between cells during the immune responses [30]. Interleukin-1β is a pro-inflammatory cytokine [31]. It plays a role in inflammatory reactions and activates macrophages and T cells [32,33,34,35]. Similar to IL-1β, IL-6 is another pro-inflammatory cytokine [36]. IFN-α is a member of interferon family and has antiviral function [37,38]. Upregulation of this primary factor of innate immunity signs viral infection of the host and involves high expressions of other genes that have role in antiviral immune response [39]. IFN-γ is another pro-inflammatory cytokine, which stimulates the macrophages and produced by Th1 cells [8,40]. Toll-like receptors are type I transmembrane proteins and perceive the occurrence of pathogen associated molecular patterns (PAMPs) and are important for innate immunity [41,42]. Toll-like receptor 4 (TLR-4) recognizes lipopolysaccharide (LPS) from Gram negative bacteria and toll-like receptor 5 (TLR-5) recognizes bacterial flagellin, a sub-unit of the bacterial flagellum [43,44]. Furthermore, bacterial TLR agonists (lipopolysaccharide and flagellin) enhance expression levels of additional pro-inflammatory cytokines as IL-1β and IL-6 [45]. Due to the correlation among toll-like receptors and pro-inflammatory cytokines, as well as the limited studies available investigating feed supplements on chicken TLRs, the mentioned immunological parameters were involved in our study.

To our knowledge, the impacts of the bioactive compounds applied in the current study have yet to be examined elsewhere in poultry. Therefore, cytokine and toll-like receptor gene expression analysis and intestinal morphometric measurements were carried out in chicken under *Escherichia coli* lipopolysaccharide challenge.

## 2. Materials and Methods

### 2.1. Ethical Approval

Experiments were confirmed by University of Debrecen Committee of Animal Welfare, Hungary (Permit number: DEMAB/12-7/2015).

### 2.2. Birds and Housing

A total of 900, 1-day old Ross 308 mixed-sex broilers were used from a commercial hatchery in Hungary. The experiment was carried out on the experimental farm of University of Debrecen, Farm and Regional Research Institute. All broilers were placed in the same barn. Chickens were kept in floor pens covered with wood shavings in a thermostatically controlled house at a stocking density of 650 cm^2^/bird. Temperature was 32 °C at placement and gradually decreased by 1.5 °C/week. The birds were exposed to light according to Olanrewaju et al. [46] as follows: 23L:1D during the first 7 days, 20L:4D between 8−28 days and 23L:1D between 29−42 days (L = light, D = dark).

### 2.3. Evaluated Parameters and Used Methods

Effect of β-glucan (positive control), carotenoid, oligosaccharide and anthocyanin supplements were examined on growth performance, such as body weight (BW), average daily gain (ADG) and average daily feed intake (ADFI). Effects of the supplements were also investigated on chicken cytokines and toll-like receptors gene expressions, such as interleukin-1β, interleukin-6, interferon-α, interferon-γ, toll-like receptor 4 and toll-like receptor 5 under *Escherichia coli* lipopolysaccharide (LPS) challenge. For gene expression analysis, total RNA was isolated with commercially available kit (Zymo Research, Orange, CA, USA) from spleen and ileum samples, RNA concentrations were measured with NanoDrop ND-1000 Spectrophotometer (Thermo Fisher Scientific, Waltham, MA, USA) and RNA integrity was checked by 1% agarose gel electrophoresis. RNA was reverse transcribed into cDNA, then qPCR reactions were carried out and results were generated using the Pfaffl method [47]. Small intestinal morphometric parameters, as villus height, crypt depth, villus height:crypt depth ratio (VH:CD ratio) and total mucosa thickness were also measured to investigate the effects of natural compounds and LPS challenge. For tissue morphology, formalin-preserved ileal tissues were used for hematoxylin-eosin stain [48], then Zeiss SteReo V8 microscope (Carl Zeiss, Jena, Germany) paired with a Zeiss camera (Carl Zeiss, Jena, Germany) was applied to capture images of stained ileal segments. Morphometric parameters were measured according to Munyaka et al. [49]. Each method is described in detail in the relevant subchapter.

### 2.4. Preparation and Determination of Extracts

#### 2.4.1. Carotenoids

**Figure 1:**
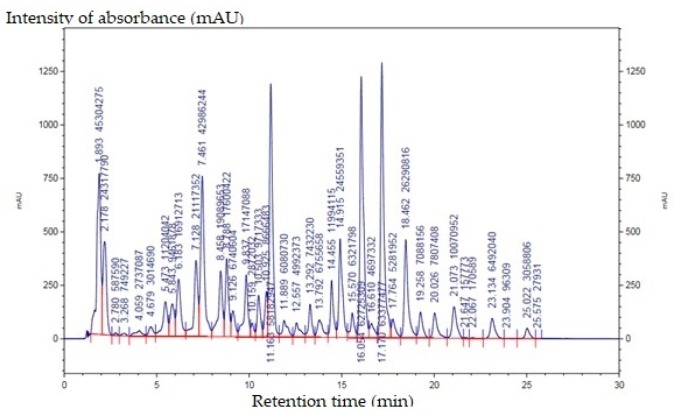
HPLC profile of DAD detection of carotenoids on 460 nm from Hungarian red sweet pepper.

Hungarian red sweet pepper powder (in 1–5 g) was applied in triplicates to extract carotenoids and extraction was carried out with 50 mL dichloroethane:acetone:methanol as solvent mixture in 2:2:1 ratio. Next, the mixture was mixed in an ultrasonic water bath for 30 min, then filtered through a filter paper (Munktell-292). Afterwards, a 0.22 µm PTFE syringe filter (TPP Techno Plastic Products AG, Trasadingen, Switzerland) was used for further purification. Filtered residue was vaporized at 40 °C at 0.2 bar and then filtrate was solved in an high-performance liquid chromatographic (HPLC) pigment reagent (isopropanol:ACN:methanol in 55:35:10 proportion) (Merck, Darmstadt, Germany) [50]. A HPLC separation was performed on Phenomenex Kinetex^®^ column (2.6 µm, XB-C18, 100 Å, 100 × 4.6 mm) (Phenomenex, Torrance, CA, USA) with 2 gradient elutions: A: 11% methanol, B: isopropanol:ACN:methanol (55:35:10 V/V/V%) mixture. Gradient elution steps were the following: 0–3 min solvent A 100%; 15–20 min solvent A 20%; 25–45 min solvent B 100%; 48–50 min solvent A 100%. Flow rate was 0.6 mL/min and Diode Array Detector (DAD) detection was carried out on 460 nm and 350 nm. Samples were injected in 10 µL and after DAD detection was applied at 460 nm and 350 nm. HPLC profile is shown in Figure 1. Carotenoid compounds with the greatest areas were identified and involved in Table 1.

#### 2.4.2. Oligosaccharides

As natural prebiotics, oligosaccharides with high arabino-galactose content were extracted from Hungarian red sweet pepper retained from industrial food waste. A HP 5890 Gas chromatograph with SP-2380 capillary column (30 m × 0.25 mm, 0.2 µm) was used to evaluate the composition of oligosaccharides. Samples were lyophilized and extracted with a trifluoracetic acid:acetic acid:water in a 5:75:20 proportion as solvent. Before identification, oligosaccharides were turned into alditol-acetate. After reduction, sugars were changed to sugar alcohols (alditols) which eliminate interfering isomers and anomers. Reduction was performed with NaBH_4_ at alkaline pH. Acetylation was also carried out with acetic anhydride in pyridine. Feed gas was nitrogen at 1.2 mL/min flow rate. Injector temperature was 300 °C, split ratio was 1:20. After Flame Ionization Detector (FID) detection, monomer units of oligosaccharides were identified and involved in Table 2.

#### 2.4.3. Anthocyanins

Anthocyanins were extracted from Hungarian sour cherry. After fruits were deseeded and homogenized, a methanol:water:acetic acid solution in 25:24:1 ratio was applied for extraction. Further processes involved mixing with Magnetic stirrer MSH 300 (BioSan, Riga, Latvia) through 1 h, filtering and centrifuging at 10,000 rpm for 5 min, then a simple fractionation was carried out in pre-conditioned tubes (Supelclean ENVI-18 SPE tubes). Tubes were conditioned with 5 mL MeOH first, then 5 mL H_2_O, then 1 mL of fruit sample was used. Elution was carried out with methanol including 20% H_2_O solution, and vaporized at 40 °C (BÜCHI ROTAVAPOR R-210, Flawil, Switzerland). Samples were dried in vacuum and anthocyanin extract reached powder formula. Determination of anthocyanin profile was carried out with VWR-Hitachi ChromasterUltraRs UHPLC (Hitachi, Tokyo, Japan) using a Phenomenex Kinetex^®^ column (2.6 µm, XB-C18, 100 Å, 100 × 4.6 mm) (Phenomenex, Torrance, CA, USA). 2 solvents were A: MeOH and B: 3% formic acid. Gradient elution steps were the following: 0 min solvent A 15%; 0–25 min solvent A 30%; 25–30 min solvent A 40%; 30–40 min solvent A 50%. Flow rate was kept at 0.7 mL/min and temperature was 25 °C. Anthocyanin composition was quantified by comparison with the corresponding authentic standards. UV-VIS detection was performed at 535 nm and injection volume was 10 µL [51]. The main anthocyanin compounds are included in Table 3 [52].

### 2.5. Experimental Design, Dietary Treatments and Growth Performance

The 1-day old Ross 308 hybrid chicken were randomly placed into 5 experimental groups (3 pens/treatment, 60 birds/pen). The experiment was started at 1 day of age and lasted until 42 days of age. The dietary treatments consisted of the control group (basal diet), the β-glucan considered as positive control and supplementation of carotenoids, oligosaccharides or anthocyanins. β-glucan was added at 0.05% to the basal diet (Table 4), additional treatments included 0.5% of bioactive extracts. The basal diets (pre-starter, starter, grower, finisher) were corn-soybean meal diets. All diets were fed in mash form. Feed and water were available ad libitum during the entire experiment. Broilers were weighed at 1, 10, 21, 32, 42 days of age. As growth performance parameters, average body weight (BW), average daily gain (ADG) and average daily feed intake (ADFI) were calculated. On day 26, 6 male chickens per treatment were injected with 2 mg/kg liveweight *Escherichia coli* O55:B5 lipopolysaccharide (LPS) (L2880, Sigma, St. Louis, MO, USA) with a concentration in 2 mg/mL, intraperitoneally. In the control group, another 6 male chickens were inoculated with 2 mL/kg liveweight isotonic saline solution (B. Braun, Budapest, Hungary) in the same way [27].

### 2.6. Sample Collection and Lymphoid Organ Weight

On day 27, 12 h after challenge, individual bodyweight of broilers was measured, then all of the injected birds (*n* = 6/treatment; control: *n* = 6/saline and *n* = 6/LPS-inoculated) were euthanized by cervical dislocation for the collection of tissue samples. Spleen and terminal ileum tissues were aseptically excised, the whole spleen was measured then samples were snap-frozen in liquid nitrogen and stored at −80 °C for RNA isolation [27]. Further 1 cm segments from the terminal sections of ileum (*n* = 3/treatment) were collected and preserved in formalin (10% neutral-buffered formalin solution, Sigma-Aldrich, Hungary) for tissue morphology [53].

### 2.7. RNA Isolation and Reverse Transcription

Total RNA from spleen and ileum tissues was extracted using Direct-zol™ RNA MiniPrep (Zymo Research, Orange, CA, USA) according to the manufacturer’s protocol, including DNA digestion step. Concentration of the RNA in each sample was measured using a NanoDrop ND-1000 Spectrophotometer (Thermo Fisher Scientific, Waltham, MA, USA). RNA integrity was checked by 1% agarose gel electrophoresis. 800 ng of the isolated RNA was reverse-transcribed using qPCR BIO cDNA Synthesis Kit (PCR Biosystems, London, UK) in 20 µL final volume containing oligo (dT)s, random hexamers and MMLV type reverse transcriptase. Conditions consisted of: reverse transcription 42 °C for 30 min and reverse transcriptase denaturation at 85 °C for 10 min. cDNA samples were diluted 10-fold and stored at −20 °C.

### 2.8. qPCR Analysis of Cytokine and Toll-Like Receptor Genes

Forward and reverse primers for chicken *IL-1β*, *IL-6*, *IFN-α*, *IFN-γ*, *TLR-4* and *TLR-5* (Table 5) were designed by Primer Express v3.0.1 software and checked for target identity using National Center for Biotechnology Information (NCBI) Primer Blast [54]. Quantitative PCR was performed by LightCycler 480 Instrument II (Roche Life Science, Penzberg, Germany), reactions were run in triplicates using 384-well plates (4titude, Surrey, UK). Each reaction included a 4 ng cDNA template, 2× Xceed qPCR SG Hi-ROX Mix (Institute of Applied Biotechnologies, Prague, Czech Republic), 200 nM of each primer and distilled water in 10 µL final volume. No template controls were included for each primer. Real Time PCR conditions were the following: initial denaturation at 95 °C for 2 min, 40 cycles of denaturation at 95 °C for 5 s and annealing/extension at 60 °C for 30 s. Raw fluorescent data were collected by LightCycler software 1.5.0 (Roche Life Science) then Ct values and main reaction efficiencies were determined with linear regression analyses on individual amplification curves by using LinReg PCR 2017.0 software. Among the most frequently used reference genes in chicken gene expression studies, stability of β-cytoskeletal actin (*ACTB*), glyceraldehyde 3-phosphate dehydrogenase (*GAPDH*) and 18S ribosomal RNA (*RN18S*) were analyzed by 3 algorithms, namely ΔCt, NormFinder, Best Keeper [55]. In spleen *GAPDH*, in ileum *ACTB* were considered as the most stabile genes for normalization. Results were generated using the Pfaffl method [47] by normalizing the expression of the target gene to a housekeeping gene. Results were determined as fold changes of the expression of the target genes in the experimental groups compared with LPS injected control group.

### 2.9. Intestine Morphometric Measurements

Formalin-preserved terminal ileal segments were used to determine villus height, crypt depth, villus height to crypt depth ratio (VH:CD ratio) and total mucosa thickness. Hematoxylin-eosin stain was carried out on 18 samples (*n* = 3 birds per treatment) [48]. Zeiss SteREO V8 (Carl Zeiss, Jena, Germany) microscope paired with a Zeiss Camera (Carl Zeiss, Jena, Germany) were used to capture images of stained segments. Photos were evaluated by Adobe Photoshop CC version 19.1.6 software. Height of villus was determined from the tip of the villus to the top of the lamina propria, the depth of the crypt was determined from the villus-crypt axis to the base of crypt and total mucosa thickness was measured from the top of the villus to the wall of intestine including villus height, crypt depth and muscolaris mucosa [49].

### 2.10. Statistical Analysis

The main effects of the bioactive compounds under immunological challenge were analyzed using One-way analysis of variance (one-way ANOVA) Tukey-test by GraphPad Prism 6.0.1 software. Differences among groups were considered significant at *p* < 0.05.

## 3. Results

### 3.1. Growth Performance

Dietary effects related to growth performance such as body weight (BW), average daily gain (ADG) and average daily feed intake (ADFI) were measured (Table 6). Among treatments, β-glucan supplementation had positive effect on BW of broilers compared to those birds in anthocyanin supplemented group on day 21, but no other treatments could affect BW compared to control treatment. ADG was not impacted by dietary treatments through the experimental period. ADFI was increased by anthocyanin supplementation under grower period (day 22–31). In addition, β-glucan, oligosaccharide and anthocyanin supplementations could increase ADFI through the experimental period (day 1–42) compared to the control.

### 3.2. Lymphoid Organ Weight

**Figure 2:**
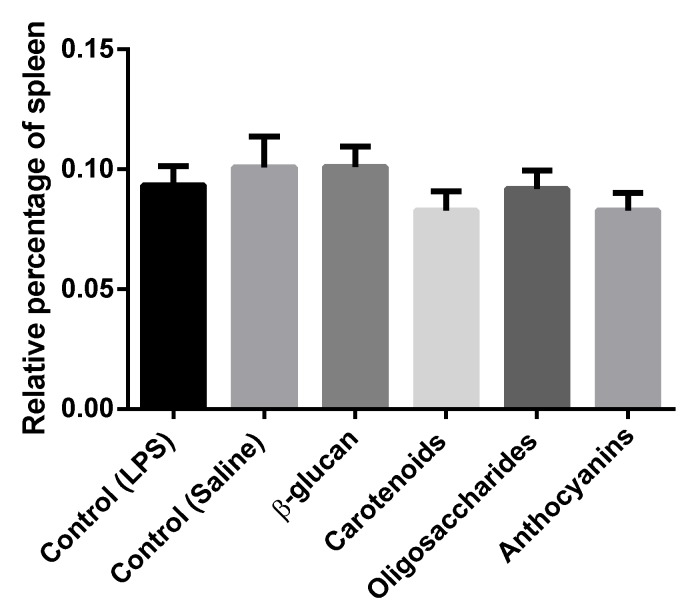
Relative spleen weight (spleen weight compared to live weight) of chickens fed basal diet under *Escherichia coli* O55:B5 LPS challenge, basal diet under isotonic saline challenge, diet supplemented with 0.05% β-glucan-, diet supplemented with 0.5 % carotenoids-, diet supplemented with 0.5% oligosaccharides- and diet supplemented with 0.5% anthocyanins under *Escherichia coli* O55:B5 LPS challenge (*n* = 6/treatment). Error bars represent means ± standard errors of the mean. The effects were analyzed by One-way ANOVA and differences among treatments were considered significant at *p* < 0.05. Dietary effects were not significant.

Relative weight of spleen compared to live weight (LW) did not show significant differences among treatments (Figure 2). Results were the following: control (saline): 0.100% of LW, control (LPS): 0.093% of LW, β-glucan: 0.100% of LW, carotenoids: 0.082% of LW, oligosaccharides: 0.091% of LW, anthocyanins: 0.082% of LW.

### 3.3. Cytokine and Toll-Like Receptor Gene Expression Analysis

**Figure 3:**
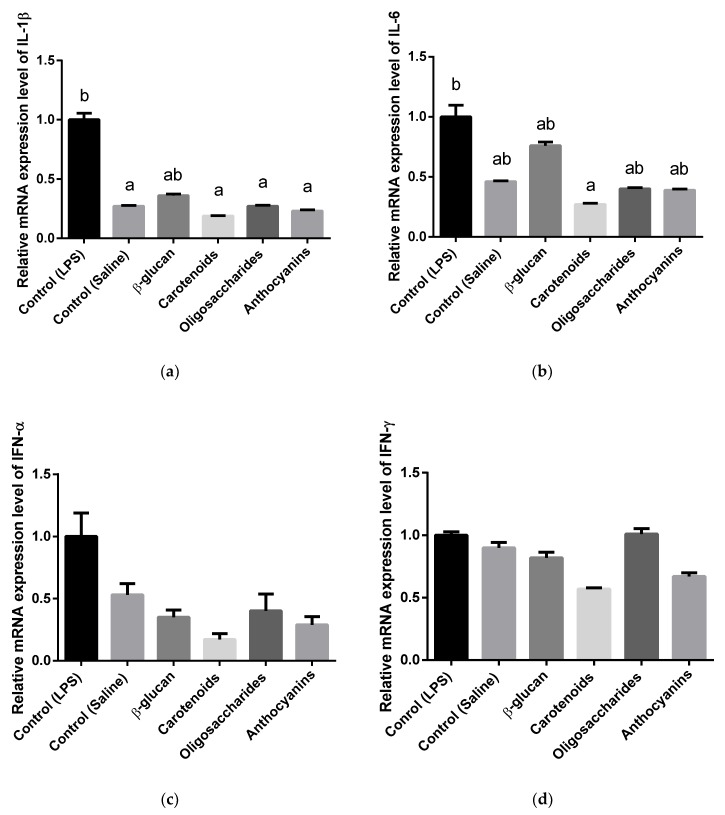

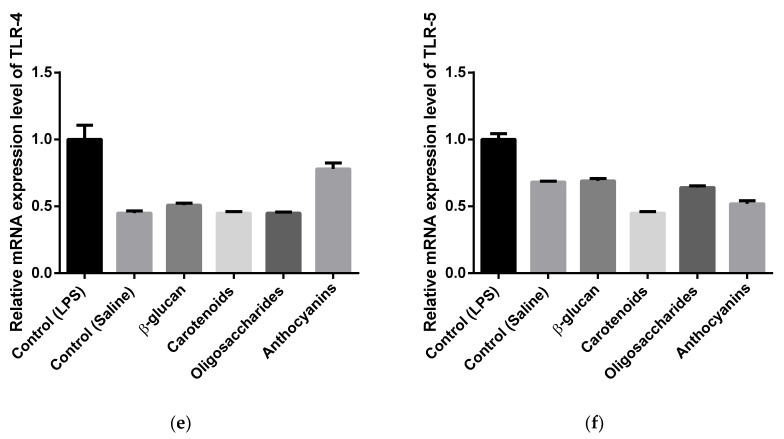
Relative interleukin-1β (**a**), interleukin-6 (**b**), interferon-α (**c**), interferon-γ (**d**), toll-like receptor 4 (**e**) and toll-like receptor 5 (**f**) mRNA expression in spleen of chickens fed basal diet under *Escherichia coli* LPS challenge, basal diet under isotonic saline challenge, diet supplemented with 0.05% β-glucan-, diet supplemented with 0.5% carotenoids-, diet supplemented with 0.5% oligosaccharides- and diet supplemented with 0.5% anthocyanins under *Escherichia coli* O55:B5 LPS challenge (*n* = 6/treatment). Error bars represent means ± standard errors of the mean. The effects were analyzed by One-way ANOVA and groups that do not share a letter are significantly different (*p* < 0.05).

Relative mRNA expression levels in spleen are shown in Figure 3. The results showed that β-glucan had no significant effect on splenic cytokine mRNA expressions of broiler chickens. Carotenoids decreased splenic IL-1β (*p* = 0.0114) and IL-6 (*p* = 0.0325) gene expressions compared to the LPS injected control group, but none of the splenic IFN-α, IFN-γ and TLR-4 were influenced by the treatment under the challenge. IL-1β gene expression was also inhibited (*p* = 0.0497) by oligosaccharide supplementation. No significant effects were observed on chicken splenic IL-6, IFN-α, IFN-γ or TLR-4 by oligosaccharide supplementation. Anthocyanins resulted in decreased IL-1β level (*p* = 0.0303), but levels of other cytokines, such as IL-6, IFN-α, IFN-γ, TLR-4, TLR-5 did not differ significantly compared to the control (LPS) group.

**Figure 4:**
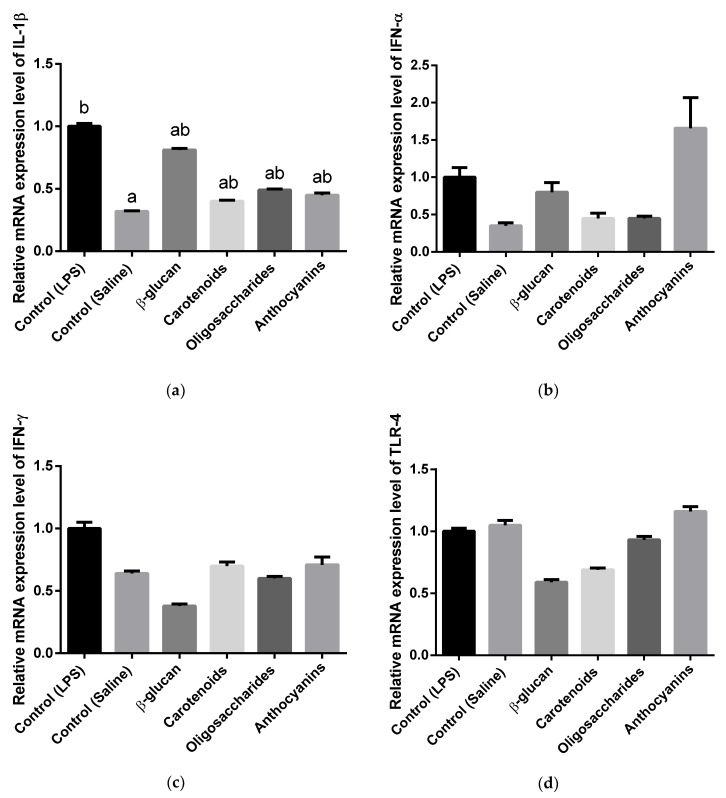

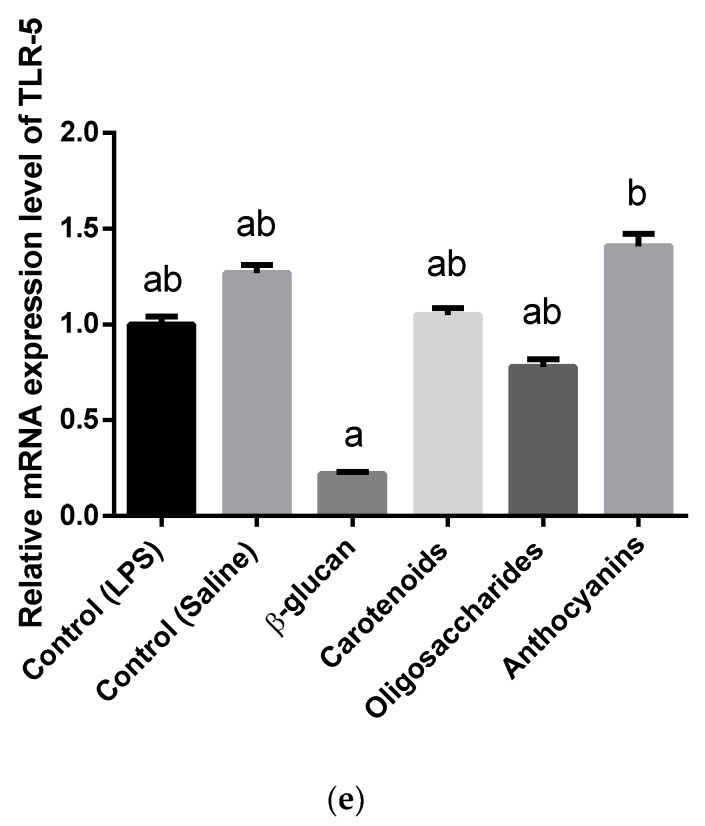
Relative interleukin-1β (**a**), interferon-α (**b**), interferon-γ (**c**), toll-like receptor 4 (**d**) and toll-like receptor 5 (**e**) mRNA expression in ileum of chickens fed basal diet under *Escherichia coli* LPS challenge, basal diet under isotonic saline challenge, diet supplemented with 0.05% β-glucan-, diet supplemented with 0.5% carotenoids-, diet supplemented with 0.5% oligosaccharides- and diet supplemented with 0.5% anthocyanins under *Escherichia coli* O55:B5 LPS challenge (*n* = 6/treatment). Error bars represent means ± standard errors of the mean. The effects were analyzed by One-way ANOVA and groups that do not share a letter are significantly different (*p* < 0.05).

Results of ileal gene expression analyses are shown in Figure 4. In the terminal part of the ileum, supplementation of β-glucan inhibited *TLR-5* gene expression (*p* = 0.0387) compared to the anthocyanin treatment, but no significant differences were observed in *IL-1β-*, *IFN-α-*, *IFN-γ-* and *TLR-4* mRNA expression levels. Effects of carotenoids or oligosaccharides were not observed on ileal interleukin-, interferon- and toll-like receptor profiles. Anthocyanins did not affect ileal cytokine mRNA expressions either, but increased ileal *TLR-5* mRNA expression was measured in anthocyanin treatment compared to β-glucan. Unlikely the spleen, *IL-6* was not expressed in the ileum. 

### 3.4. Intestine Morphometric Measurements

Ileal villus height was significantly higher in treatments of β-glucan (*p* < 0.0001), carotenoid (*p* < 0.0001), oligosaccharide (*p* < 0.0001) and anthocyanin (*p* < 0.0001) (Table 7). Height of villi did not differ significantly in control groups (*p* = 0.212). Significantly higher crypt depth was measured in oligosaccharide supplementation (*p* < 0.0001). Depth of crypt was lower in saline inoculated control group (*p* = 0.0009) compared to LPS inoculated birds. Crypt depth did not vary in β-glucan and anthocyanin treatments. Villus height to crypt depth (VH:CD) ratio was higher in β-glucan (*p* < 0.0001) and anthocyanin (*p* = 0.0063) supplementations contrasted to lipopolysaccharide injected control group. No difference in VH:CD ratio was observed in control-saline, carotenoid- and oligosaccharide supplemented diets. Higher total mucosa thickness of ileum was observed in β-glucan (*p* < 0.0001), oligosaccharide (*p* < 0.0001) and anthocyanin *(p* = 0.048) supplementations, but no variations were found in control-saline and carotenoid groups.

## 4. Discussion

Experiments were conducted to examine the effects of four bioactive compounds on growth performance, spleen weight, immunological parameters and intestinal morphology. The effect of feed supplemented with carotenoids, oligosaccharides, anthocyanins using *Escherichia coli* lipopolysaccharide challenge was investigated BW, ADG and ADFI of broiler chicken. None of the treatments impacted BW of chickens compared to control birds, but β-glucan could significantly impact the BW of broilers on day 21, compared to anthocyanin supplementation. Zhang et al. [7] proved the same, when β-1,3/1,6-glucan supplementations in 50 and 75 mg/kg could increase BW of chicken. None of the compounds applied in this study could increase ADG of chickens. Similarly, Rezaei et al. [56] defined no significant differences in ADG of broilers, when diet was supplemented using 0.5% and 1% oligosaccharides extract, which could have been due to the low concentration of the used supplementation. Thus, the growth and activity of beneficial bacteria could not have been promoted. In this study, anthocyanin supplementation increased ADFI between days 22–31, and β-glucan, oligosaccharides and anthocyanins additionally affected ADFI positively through the whole experimental period (day 1–42). Similarly, Zhang et al. [7] measured enhanced feed intake of chickens after 50 and 75 mg/kg β(1-3)(1-6)-d-glucan feed supplementations were used. Rezaei et al. [56] also reported altered animal production during the finisher period and through the entire experiment (day 1–35) after 1% oligosaccharide addition to diet, so higher feed efficiency was observed at oligosaccharide-fed birds compared to control ones.

Effects of mentioned supplementations were also examined on the weight of chicken spleen. Relative weight of spleen of broiler chickens were not different among diets supplemented with bioactive compounds. A similar result was reported by Shang et al. [27] who discussed healthy immune status of broiler chickens, when relative weights of spleen and bursa of the Fabricius of chicken did not differ significantly on day 21 when diet was supplemented with fructooligosaccharide and chickens challenged with *Salmonella enteritidis* lipopolysaccharide. Rathgeber et al. [57] also found that when diet was supplemented with β-glucan was not affected broiler spleen weights compared to body weight between the treatment and control groups on experimental days 14 and 38. Wang et al. [13] also reported live yeast addition did not affect spleen weights of broilers. Kamboh et al. [58] measured higher spleen indices (*p* < 0.01) in broilers under LPS challenge on day 21 when diet was supplemented with bioflavonoids (genistein and hesperidin = 1:4, 20 mg/kg), although spleen indices were not influenced when genistein and hesperidin were used at lower concentrations or alone.

β-glucan was applied as a positive control in this study, whereas previous ones reported it could maintain a beneficial immune status in chickens under a microbial challenge [59] and found β(1-3)(1-6)-d-glucan could reduce pro-inflammatory TNF-α and cortisol levels in serum. Another study showed yeast cell wall could increase the cutaneous hypersensitivity reaction, which was explained as an indirect factor in cellular immune response and a positive effect of yeast cell wall was defined [60]. Shen et al. [61] reported elevated effect of *Saccharomicces cerevisiae* supplementation in pigs that resulted in a low pro-inflammatory IFN-γ level in serum. Live yeast also reduced serum IL-1β level in pigs under LPS challenge [12]. Wang et al. [13] highlighted live yeast (*S. cerevisiae*) improved immune status of broilers infected with lipopolysaccharide by reduction of inflammatory interleukin-1β. In our study β-glucan neither have any effect on chicken splenic or ileal cytokines and receptors (*IL-1β*, *IL-6*, *IFN-α*, *IFN-γ*, *TLR-4*, *TLR-5*) compared to LPS-injected control birds. Similarly, Markazi et al. [9] found yeast cell wall products could not affect inflammatory *IL-1β* mRNA expression in chicken cecal tonsils under a coccidial challenge. Kumar et al. [62] also discussed that β-glucan treatment did not produce different expressions of *IL-1β*, *TLR-4* and *TLR-5* in chicken spleen compared to LPS challenged birds. However, this study shows β-glucan supplementation could decrease the ileal gene expression level of *TLR-5* compared to anthocyanin-fed birds. Sheoran et al. [63] explained reduced *TLR-5* mRNA expression with the decreasing colonization of pathogens and Shanmugasundaram et al. [64] reported whole yeast cell product supplementation could influence the growth of beneficial bacteria, such as *Lactobacillus* and *Bifidobacterium*. Therefore, downregulation of *TLR-5* mRNA level in β-glucan treatment could have been due to the same fact in this study.

The effect of carotenoid supplementation was also investigated on chicken immune cytokines. As we predicted, both splenic and ileal *IL-1β* gene expression levels were high in LPS-injected birds compared to saline-inoculated controls, whereby *Escherichia coli* lipopolysaccharide could induce an acute immune response and a bacterial illness [8]. Munyaka et al. [49] found the same and reported higher *IL-1β* gene expression levels in spleens of lipopolysaccharide-injected chickens compared to saline-inoculated ones. In our study, carotenoids could inhibit splenic *IL-1β* gene expression, though it did not influence the ileal *IL-1β* gene expression level compared to lipopolysaccharide-treated control birds. Gao et al. [19] also examined the impact of xanthophyll—a class of carotenoids—on chicken pro-inflammatory cytokines in ileum and reported no significant effects of 20 or 40 mg/kg xanthophyll supplementation. Similar to IL-1β, up-regulation of pro-inflammatory IL-6 can be explained as an acute-phase reaction [65]. This study shows a high-expression of interleukin-6, and carotenoids could reach low gene expression level of *IL-6* in the spleen. These results suggest carotenoids are useful in reducing the effect of inflammation through decreasing inflammatory parameters. Shanmugasundaram and Selvaraj et al. [66] discussed the same, when lutein supplementation decreased pro-inflammatory *IL-1β* gene expression in turkeys under LPS challenge.

The impact of oligosaccharides with high arabino-galactose content were also investigated in this study. Applied oligosaccharides could reach a low gene expression level of pro-inflammatory *IL-1β* in spleen, which result shows oligosaccharides can be also effective in mitigating the inflammation. It could not influence mRNA expression level of *IL-1β* in ileum.

The effects of anthocyanins were also examined on chicken immune response. Our research shows anthocyanins could reduce the amount of *IL-1β* mRNA in chicken spleen. Changxing et al. [29] also studied the effect of anthocyanins and described anthocyanin-supplementation could reduce cyclo-oxygenase-1 (COX-1) and cyclo-oxygenase (COX-2) inflammatory enzymes, which could inhibit expressions of pro-inflammatory interleukins. Anthocyanin-rich fragment was also applied by Li et al. [28] and it could prevent *IL-1β* mRNA expression in mice, similarly. These findings suggest the anti-inflammatory effect of anthocyanins confirmed by Carvalho et al. [67] when anthocyanin supplementation decreased the level of pro-inflammatory cytokines (IL-1β, IL-6, IFN-γ) in ethidium-bromide induced rats. Effect on ileal interleukin-1β gene expression level was not observed in our study.

Except carotenoids, no other compounds could influence splenic *IL-6* gene expression. Accordingly, a study conducted to analyze the effect of galactoglucomannan oligosaccharide-arabinoxylan had no effect on mRNA expression of *IL-6* in chicken spleen [68]. However, xylooligosaccharides at 2 g/kg in diet could reach a low *IL-6* mRNA expression in cecal tonsils of chicken under *Salmonella* challenge [69]. In addition, none of carotenoids, oligosaccharides or anthocyanins influenced gene expression levels of splenic and ileal toll-like receptor 4 (*TLR-4*), ileal toll-like receptor 5 (*TLR-5*), splenic and ileal interferon-α (*IFN-α*) and splenic interferon-γ (*IFN-γ*) in the recent study. In our study, higher relative expression level of *TLR-4* was expected in the control treatment that involved LPS challenge compared to control-saline birds, since Tan et al. [70] measured higher *TLR-4* gene expression in spleen, when chickens were injected with LPS. In the same study, authors defined decreased *TLR-4* expression when dietary supplementation was applied. Nevertheless, no significant differences were observed in spleen or ileum in our study. Similarly, Kumar et al. [62] identified that β-glucan in 0.10% could not alter *TLR-4* mRNA expression in chicken spleen; however, no LPS challenge was involved in that study. Gene expression level of *TLR-5* in this study was not altered significantly among the treatments. In contrast with our result, Sheoran et al. [63] experienced the down-regulation of *TLR-5* mRNA in chicken blood, when mannan-oligosaccharide-based prebiotic product was applied at 0.5, 1 and 2 g/kg in the diet. Therefore, the impact of mannan-oligosaccharides in that study was discussed as it was capable of reducing colonization of Gram-negative pathogens, as well as those involving flagellin, such as *E. coli* or *Salmonella spp*. In this case, no significant differences were detected in *IFN-α* gene expression levels. In contrast, Khan et al. [71] studied higher *IFN-α* gene expression in spleen of laying hens under Infectious Bronchitis Virus (IBV) T strain challenge, so viral RNA was recognized and immune system of hens activated the defense mechanisms. Significant differences in splenic or ileal *IFN-γ* expressions were not measured among treatments. In contrast, Li et al. [72] showed increased *IFN-γ* gene expression level in spleen of chicken under LPS challenge. Cox et al. [73] also reported higher *IFN-γ* expression in intestinal segments of chicken challenged with *Eimeira* oocysts and expression level of mentioned cytokine was down-regulated by β-glucan supplementation. Pourabedin et al. [69] reported mannan-oligosaccharides supplementation in 1 g/kg in diet could inhibit the gene expression level of pro-inflammatory *IFN-γ* in chicken cecal tonsils under *Salmonella enteritidis* challenge and discussed that *IFN-γ* enhances the activation of macrophages and the production of nitric oxide.

Intestinal morphological measurements were also carried out to define changes in terminal ileum tissues which can refer to digestive functions. Increased villus heights and decreased depth of crypt can provide a larger surface for digestion and absorption of nutrients [49]. In our study, β-glucan, carotenoid, oligosaccharide and anthocyanin supplementation influenced positively the length of villus in terminal ileum segments which can point to a beneficial effect in absorption functions. Among treatments, oligosaccharides increased the depth of the crypt in the ileum. No other alterations were observed on crypt depth except in ileum of saline-injected birds where shorter crypt depth was measured, in contrast to the intestinal segments of LPS inoculated birds. Higher villus height to crypt depth ratios (VH:CD) were shown only in the β-glucan and anthocyanin treatment. Diets supplemented with β-glucan, oligosaccharide and anthocyanin thickened the mucosa. These findings indicate an increased absorption area in the ileum of treated birds. Similarly to our results, villus height, crypt depth and total mucosa thickness were significantly higher after 0.5% fructooligosaccharide supplementation in the diet of chickens challenged with *Escherichia coli* LPS [27]. Xu et al. [26] reported the same, when 0.4% fructooligosaccharide supplementation resulted in higher villus length and VH:CD ratio in ileum in broilers. Shanmugasundaram et al. [74] also reported higher VH:CD ratios in chicken fed yeast cell wall supplemented diet.

## 5. Conclusions

In conclusion, bioactive compounds, such as β-glucan, carotenoids, oligosaccharides and anthocyanins, could partially affect growth performance, inflammatory parameters and intestinal morphology in broilers under LPS challenge. β-glucan can be useful for improving the body weight of chickens, for maintaining beneficial microflora by eliminating pathogens and increasing the ileal absorption surface by influencing villus height, VH:CD ratio and total mucosa thickness. Carotenoids are being suggested to decrease inflammation through an acute phase response and to increase the villus length. Oligosaccharides could also alleviate an inflammatory parameter and additionally affect morphometric factors by improving villus length and mucosa thickness. Anti-inflammatory effect of anthocyanins is also proved by decreasing inflammation and effective absorption functions are also indicated by longer villi, increased VH:CD ratio and thickened mucosa.

## Figures and Tables

**Table 1:** Identified carotenoid compounds with relative percentage of areas.

Retention Time	Name of Compound	Relative Percentage of Areas (%)
11.163	β-carotene	9.965
16.054	cis-capsanthin	10.743
17.17	capsanthin	10.854
18.462	zeaxanthin	4.503

**Table 2:** Identified oligosaccharide monomers with relative percentage of areas.

Name of Monomers	Relative Percentage of Areas (%)
Glucose	71.310
Arabinose	8.993
Xylose	8.697
Galactose	6.815
Mannose	4.185

**Table 3:** Identified anthocyanin compounds [52].

Name of Anthocyanin Compounds	Quantity (mg/100 g)
cyanidin-3-*O*-glucosyl-rutinoside	2.77–10.31
cyanidin-3-*O*-rutinoside	4.93–14.56
cyanidin-3-*O*-monoglucoside	2.02–7.79

**Table 4:** Composition and nutrient level of the basal diets.

Basal Ingredients	Value			
	Pre-Starter (Day 1–9)	Starter (Day 10–21)	Grower (Day 22–31)	Finisher (Day 32–42)
Corn, %	33	34	33	32
Wheat, %	27	29	31	32
Soybean meal, solvent extracted (46.0% CP), %	29	24	20	16
Soybean meal, extruded (46.0% CP), %	4	6	4	4
Sunflower meal, extracted, %		1	3	4
Feed yeast, %	1			
DDGS, %		1	3	5
Plant fats, %	2	1	3	4
Premix, %	4	4	3	3
Total, %	100	100	100	100
Nutrient Level				
Dry matter, %	89.06	89.03	89.15	89.15
AME_n_ poultry, MJ/kg	12.23	12.47	12.81	13.01
Crude protein, %	21.58	20.28	19.05	18.28
Crude fat, %	4.61	4.83	6.22	6.83
Crude fibre, %	3.37	3.51	3.7	3.88
Lysine, %	1.37	1.27	1.17	1.09
Methionine, %	0.57	0.54	0.53	0.49
Methionine + Cysteine, %	0.94	0.9	0.87	0.83
Calcium, %	0.85	0.73	0.71	0.67
Phosphorus, %	0.63	0.55	0.52	0.49

**Table 5:** Primer sequences of chicken cytokines.

Accesion No. or Reference	Primer Sequences (5′→3′)	Gene	Amplicon Length (bp)	Annealing Temperature (°C)
XM_015297469.1	F: TGCTTCGTGCTGGAGTCACCC	*IL-1β*	98	59.93
	R: GGCCGGTACAGCGCAATGTT			59.02
XM_015281283.2	F: AGCGAAAAGCAGAACGTCGAGTC	*IL-6*	107	58.73
	R: GCCGAGTCTGGGATGACCACTTC			59.94
AM049251.1	F: ACTTCAGCTGCCTCCACACCTT	*IFN-α*	92	59.14
	R: CAGGAACCAGGCACGAGCTT			57.74
NM_205149.1	F: AACAACCTTCCTGATGGCGTGA	*IFN-γ*	89	57.46
	R: GCTTTGCGCTGGATTCTCAAGT			57.02
NM_001030693.1	F: ACCCGAACTGCAGTTTCTGGAT	*TLR-4*	120	57.20
	R: AGGTGCTGGAGTGAATTGGC			55.61
XM_025148815.1	F: ATGAGCTGAGGCTTTAGTTGGAGA	*TLR-5*	108	56.61
	R: CCAGCTAGTGCTATTCCAAAGACA			55.62
[55]	F: GCTGGCATTGCACTGAATGAC	*GAPDH*	113	55.73
	R: CACTCCTTGGATGCCATGT			52.42
[55]	F: AGATCACAGCCCTGGCACCTAG	*ACTB*	61	58.80
	R: TTGCGCTCAGGTGGGGCAAT			60.22

**Table 6:** Effect of natural compounds on growth performance of broiler chickens.

Diet						
Parameters	Control	β-Glucan	Carotenoids	Oligosaccharides	Anthocyanins	RMSE *
BW (g/bird)						
Day 1	38.9	37.9	38.6	38.6	38.5	0.5
Day 10	232	226	221	222	227	14
Day 21	759 ^a,b^	795 ^b^	769 ^a,b^	715 ^a,b^	726 ^a^	38
Day 32	1713	1767.7	1709.9	1735.3	1705.3	66
Day 42	2758	2727	2748	2618	2590	98
ADG (g/day/bird)						
Pre-starter (Day 1–9)	19	19	18	18	19	1
Starter (Day 10–21)	47.9 ^a,b,c^	51.8 ^c^	49.8 ^a,c^	44.9 ^b^	45.3 ^a,b^	3
Grower (Day 22–31)	87	88	86	93	89	5
Finisher (Day 32–42)	104	96	104	88	89	10
Day 1–42	65	64	65	61	61	2
ADFI (g/day/bird)						
Pre-starter (Day 1–9)	4	3	3	4	5	2
Starter (Day 10–21)	50	58	58	56	61	15
Grower (Day 22–31)	130 ^a^	144 ^a,b^	148 ^a,b^	149 ^a,b^	159 ^b^	16
Finisher (Day 32–42)	114	132	124	127	132	15
Day 1–42	73 ^a^	83 ^b^	81 ^a,b^	82 ^b^	88 ^b^	6

* Root mean square error; BW and ADG is based on individual values (*n* = 18), ADFI is calculated for pens (*n* = 3); ^a,b,c^ Mean values within a row with different superscript letters are significantly different (*p* < 0.05).

**Table 7:** Effect of natural compounds on ileum morphology of broiler chickens at 27 days of age.

Ileum Morphology	Diet						SEM	*p*-Value
	Control (LPS)	Control (Saline)	β-Glucan	Carotenoids	Oligosaccharides	Anthocyanins		
Villus height (µm)	774.31 ^a^	712.02 ^a^	998.93 ^b^	908.94 ^b^	977.08 ^b^	921.84 ^b^	27.42	<0.0001
Crypt depth (µm)	140.38 ^b,c^	107.31 ^a^	120.43 ^a,b^	160.27 ^c^	179.90 ^d^	134.47 ^b^	8.086	<0.0001
VH:CD ratio	5.83 ^a,b^	6.81 ^b,c^	8.57 ^d^	6.19 ^a,b,c^	5.70 ^a^	7.12 ^c^	0.3625	<0.0001
Total mucosa thickness (µm)	1156.89 ^a,c^	1137.47 ^a^	1350.07 ^b^	1251.06 ^b,c^	1346.49 ^b^	1286.38 ^b^	35.63	<0.0001

Mean values with their standard errors, *n* = 3/treatment; ^a,b,c,d^ Mean values within a row with different superscript letters are significantly different (*p* < 0.05) Ileal villus height was significantly higher in treatments of β-glucan (*p* < 0.0001), carotenoid (*p* < 0.0001), oligosaccharide (*p* < 0.0001) and anthocyanin (*p* < 0.0001). Height of villi did not differ significantly in control groups (*p* = 0.212). Significantly higher crypt depth was measured in oligosaccharide supplementation (*p* < 0.0001). Depth of crypt was lower in saline inoculated control group (*p* = 0.0009) compared to LPS inoculated birds. Crypt depth did not vary in β-glucan and anthocyanin treatments. Villus height to crypt depth (VH:CD) ratio was higher in β-glucan (*p* < 0.0001) and anthocyanin (*p* = 0.0063) supplementations contrasted to lipopolysaccharide injected control group. No difference in VH:CD ratio was observed in control-saline, carotenoid and oligosaccharide supplemented diets. Higher total mucosa thickness of ileum was observed in β-glucan (*p* < 0.0001), oligosaccharide (*p* < 0.0001) and anthocyanin (*p* = 0.048) supplementations, but no variations were found in control-saline and carotenoid groups.
